# Multimodal Deep Learning Approaches for Lung Disease Detection: A Review

**DOI:** 10.3390/medicina62071223

**Published:** 2026-06-24

**Authors:** Bastian Estay Zamorano, Ali Dehghan Firoozabadi, Pablo Adasme, Wanda Montiel Piña, Mauricio Chávez Muñoz, David Zabala-Blanco, Pablo Palacios Játiva, Cesar A. Azurdia-Meza

**Affiliations:** 1 Department of Electricity, Universidad Tecnológica Metropolitana, Santiago 7800002, Chile; bastian.estayz@utem.cl (B.E.Z.); wanda.montielp@utem.cl (W.M.P.); mauricio.chavezm@utem.cl (M.C.M.); 2 Department of Electrical Engineering, Universidad de Santiago de Chile, Santiago 9170124, Chile; 3 Department of Computing and Industries, Universidad Católica del Maule, Talca 3466706, Chile; dzabala@ucm.cl; 4 Escuela de Informática y Telecomunicaciones, Universidad Diego Portales, Santiago 8370190, Chile; pablo.palacios@mail.udp.cl; 5 Department of Electrical Engineering, Universidad de Chile, Santiago 8370451, Chile; cesarazurdia@uchile.cl

**Keywords:** deep learning, lung diseases, medical diagnosis, image processing, acoustic signals, multimodal sensing, biomedical sensors, respiratory sound analysis, convolutional neural networks, self-supervised learning

## Abstract

Lung diseases are among the leading global causes of morbidity and mortality, and existing reviews on deep learning (DL) for pulmonary diagnosis rarely integrate imaging, acoustic, and electronic health record (EHR) modalities within a single framework. We aimed to synthesize the state of the art (2019–2024) in multimodal DL for lung disease detection and classification, identifying dominant architectures, performance benchmarks, and translational barriers across chest X-rays, CT scans, respiratory sounds, and EHRs. A structured narrative review was conducted using PubMed, Scopus, IEEE Xplore, and Web of Science, applying explicit inclusion criteria for peer-reviewed studies; performance metrics, dataset characteristics, and reported limitations were extracted. Research involving convolutional neural networks (CNNs) and more recent models such as Transformers have reported high performance in chest X-ray classification, whereas acoustic approaches based on spectrograms and self-supervised representations (e.g., Wav2Vec 2.0) show promising but dataset-dependent results.

## 1. Introduction

Globally, most of the burden of pulmonary morbidity and mortality is attributed to four major respiratory diseases: chronic obstructive pulmonary disease (COPD), pneumonia, COVID-19, and lung cancer.

First, the World Health Organization (WHO) ranks COPD as the fourth leading cause of death worldwide, with approximately 3.5 million deaths recorded in 2021. Although it is an irreversible condition driven primarily by smoking and air pollution, its symptoms can be mitigated through smoking cessation, infection prevention, and pulmonary rehabilitation [1].

On the other hand, pneumonia stands out as the leading respiratory cause of death in children under five, accounting for almost 15% of deaths in this group during 2017 [2]. In the realm of emerging diseases, COVID-19, declared a public health emergency in 2020, was the cause of death for millions of people and exposed the vulnerability of health systems to the rapid spread of respiratory illnesses [3].

Lung cancer remains the deadliest malignant neoplasm in absolute terms, causing approximately 1.8 million deaths annually, which accounts for 18% of global cancer mortality (GLOBOCAN 2020 [4]). Although tobacco use is its primary cause, a significant proportion of cases occur in non-smoking patients exposed to environmental factors such as secondhand smoke, radon, asbestos, or air pollution [4]. Prognosis is strongly stage-dependent: five-year survival ranges from 60% at stage I to just 6% at stage IV, underscoring the imperative of accurate early-stage diagnosis [5].

Although the standard physical examination provides specific diagnostic findings, such as hyperresonance in COPD or reduced chest expansion in restrictive lung diseases, its main weakness lies in its subjectivity. Since the results depend on individual skill, it is common to find inconsistencies between the diagnoses of different physicians, even when they are evaluating the same physical findings [6].

This medical consensus gap is exacerbated by the ambiguity of physiological signals, which can lead to errors for several reasons. It is estimated, for example, that up to 25% of healthy individuals present adventitious sounds, while nearly identical sounds can be associated with pathologies as disparate as asthma or COPD [7,8]. In some contexts, stethoscope sensitivity barely reaches 29% [9]. Certain factors, such as obesity or the terminal stages of an illness, can produce the phenomenon of “silent lung.” In these situations, the lack of noise causes respiratory obstruction, so that airflow is insufficient even to generate any sound [10].

While some individual techniques offer limited accuracy, modern medicine has increasingly adopted a multimodal approach that combines information from multiple sources to achieve a more reliable diagnosis. Within this framework, deep learning has transformed medical analysis through advanced data fusion architectures. Vision–language models (VLMs), such as GPT-4V and LLaVA, employ cross-attention mechanisms to connect image data with clinical text. This enables the simultaneous interpretation of a chest X-ray alongside the patient’s electronic health record [11]. At the same time, contrast-learning-based methods, such as CLIP, and masked autoencoders (MAEs) improve the identification of latent patterns in large, unlabeled datasets, optimizing model performance compared to previously unknown cases.

The benefits of this multimodal transition are evident in clinical practice. In COPD diagnosis, combining CT-derived deep learning features with radiomics features and epidemiological questionnaire variables improved diagnostic performance over single-feature models, raising the AUC from 0.844 (deep learning alone) to 0.952 for the fusion model and 0.971 for the comprehensive model including questionnaire data [12]. In obstructive lung diseases, cross-referencing breath sounds with spirometry results allows for data classification with 92% accuracy [13]. The use of these tools in a daily hospital setting depends on solutions to the following problems: machine learning to correct population biases [14,15], and code optimization to achieve response times of less than two 69 s in the emergency department [16]. Interoperability standards such as HL7/FHIR 70 ensure that this technology integrates seamlessly into hospital information systems.

According to our literature review, no recent review has systematically examined the integration of multimodal fusion approaches, which combine imaging, respiratory acoustics, and electronic health record (EHR) data, within a timeframe between 2019 and 2024. To clarify this claim: Galic’ et al. [17] surveyed convolutional architectures across multiple medical imaging domains (breast, brain, retina, cardiovascular) without restriction to pulmonary medicine and without covering acoustic or EHR modalities. The work by Ahmed et al. [18] focused narrowly on data augmentation strategies for three-dimensional CT lung cancer prediction, constituting a single-modality, single-disease contribution rather than a cross-modal synthesis. Riquelme and Akhloufi [19] concentrated on Transformer-based feature encoders for respiratory sound classification, omitting imaging and clinical record integration entirely. Finally, Tabik et al. [20] and Chowdhury et al. [21] were restricted to COVID-19 diagnosis from chest X-rays, covering one disease episode and one imaging modality. None of these works simultaneously addresses imaging, respiratory acoustics, and EHR data across the full spectrum of major pulmonary pathologies (COPD, pneumonia, lung cancer, and COVID-19), which is the scope of the present review. This article aims to fill this gap in the literature by establishing the state of the art in multimodal deep learning applied to respiratory diagnostics and addressing three key points: (i) the lack of standardization in fusion techniques for heterogeneous data streams (imaging, respiratory acoustics, and EHR); (ii) the absence of consistent clinical validation metrics for major respiratory diseases (COPD, pneumonia, lung cancer, and COVID-19); and (iii) the need to chart a translational roadmap to convert these technical innovations into tools for real-world clinical use.

**Figure 1:**
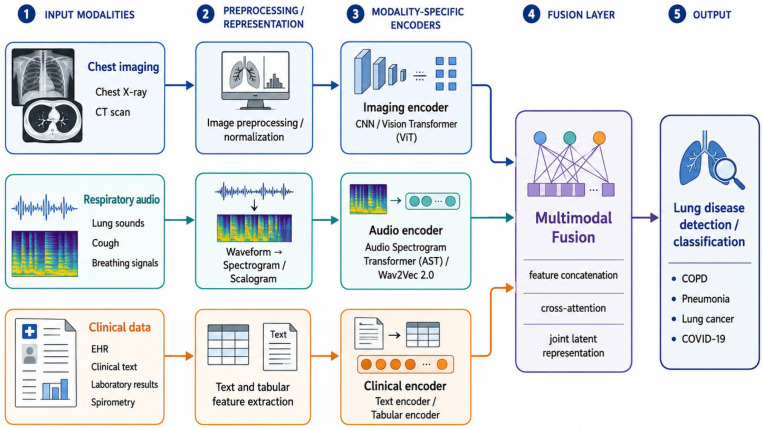
Conceptual multimodal fusion pipeline for lung disease detection. Chest imaging, respiratory audio, and clinical data are first preprocessed and encoded using modality-specific architectures, such as CNNs/ViTs for imaging, AST/Wav2Vec 2.0 for audio, and text/tabular encoders for electronic health records. The extracted features are then integrated through a multimodal fusion module, enabling improved classification of major pulmonary diseases, including COPD, pneumonia, lung cancer, and COVID-19.

To illustrate the central multimodal concept that structures this review, Figure 1 presents a conceptual workflow for lung disease detection that integrates imaging, respiratory acoustics, and clinical data through modality-specific encoders and a fusion module.

The article is organized into six main sections. Section 1 provides context on the burden of respiratory diseases and the potential of artificial intelligence. Section 2 details the search strategy and literature selection criteria. Section 3 explains the adaptation of core architectures such as convolutional neural networks (CNNs), Transformers, and autoencoders to respiratory data. Section 4 analyzes publicly available datasets and their inherent challenges, such as demographic biases and class imbalance. Subsequently, in Section 5, the evidence is compared and its limitations discussed with previous reviews. The final section, Section 6, summarizes the most impactful lines of research and proposes translational roadmaps to facilitate their future clinical implementation.

## 2. Review Methodology

This work was conducted as a structured narrative review, inspired by SPAR-4-SLR and PRISMA reporting principles, but without the full statistical pooling of a systematic review. The objective was to map and critically synthesize the state of the art in multimodal deep learning for lung disease detection rather than to perform a quantitative meta-analysis.

Search strategy: Four bibliographic databases were queried—PubMed, Scopus, IEEE Xplore and Web of Science. The search combined terms related to the application domain (lung disease, pulmonary diagnosis, COPD, pneumonia, lung cancer, COVID-19), the data modality (chest X-ray, CT, respiratory sound, auscultation, electronic health record), and the methodological family (deep learning, convolutional neural network, transformer, self-supervised learning, multimodal fusion). Boolean operators and field-specific filters were used in each database according to its syntax [S1: Supplementary Materials].

Inclusion criteria: Peer-reviewed journal articles and conference proceedings published between January 2019 and December 2024, written in English, reporting deep learning models applied to lung disease detection, classification, or segmentation from at least one of the modalities of interest (CXR, CT, respiratory sound, EHR). Foundational pre-2019 references (e.g., methodological papers cited by included studies) were retained when essential for context. Exclusion criteria: Studies focused exclusively on non-pulmonary pathologies, opinion pieces without methodological detail, predatory journal publications, and duplicate reports. Preprints (medRxiv, arXiv) were considered only when no peer-reviewed equivalent was available and were flagged as such.

Selection and synthesis process: In the first phase, records were evaluated based on their title and abstract, followed by a full-text review. Once selected, the studies were classified according to three key criteria: (i) the type of data (image, acoustic, or multimodal); (ii) the model architecture family (CNN, Transformers, autoencoders, or hybrid approaches); and (iii) the clinical objective (COPD, pneumonia, lung cancer, COVID-19, or multi-pathology analysis). Specific data were extracted from each included study, such as the dataset used, sample size, architecture, key performance metrics (accuracy, AUC, F1 score, and sensitivity), and limitations reported by the authors. All this information were synthesized narratively and supplemented with comparative tables. Finally, given the non-systematic nature of this review, no protocol was pre-registered, nor was a meta-analytic pooling of the data performed; these conditions are duly discussed as limitations in Section 5.

**Figure 2:**
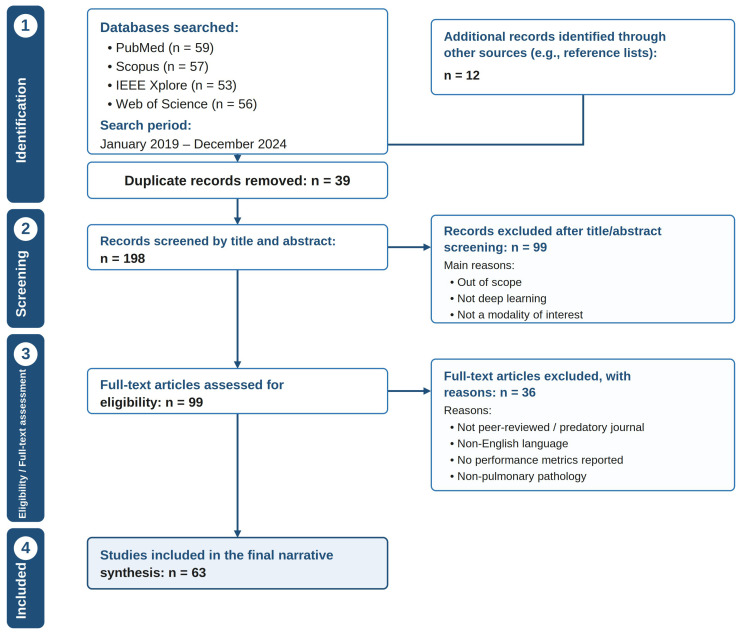
PRISMA-style flow diagram of the study-selection process, showing the number of records identified through database searching and additional sources, screened, assessed for full-text eligibility, and included in the narrative synthesis, together with the main reasons for exclusion at each stage.

The database search retrieved 225 records in total: PubMed (*n* = 59), Scopus (*n* = 57), IEEE Xplore (*n* = 53), and Web of Science (*n* = 56). An additional 12 records were identified through reference-list screening, yielding 237 records before de-duplication. After removing 39 duplicates, 198 records were screened by title and abstract; 99 were excluded at this stage (main reasons: out of scope, not a deep learning study, or not involving a modality of interest). The remaining 99 records were assessed for full-text eligibility; 36 were subsequently excluded (not peer-reviewed or predatory journal, non-English language, no performance metrics reported, non-pulmonary pathology). A total of 63 studies were included in the final narrative synthesis [S2: Supplementary Materials]. The complete selection cascade is illustrated in Figure 2.

Figure 2 summarizes the study-selection process following the PRISMA flow-diagram format, including the number of records identified, screened, assessed for eligibility, and included, together with the main reasons for exclusion at each stage.

## 3. Fundamentals of Deep Learning for Pulmonary Diagnosis

In the field of medical imaging and diagnostics, deep learning has proven to be a powerful tool. When combined with artificial intelligence (AI), it yields promising results, ranging from image reconstruction to comprehensive image analysis. As a result, the outcomes have come to rival, or even surpass, those of professional physicians in the analysis of medical images.

One of the key functions of deep learning in medical imaging is closely tied to its ability to extract features and recognize patterns in these images. For example, in Ke Song’s work [22], deep learning is applied to ultrasound images to assess the network’s potential to improve image reconstruction, reduce computational complexity, and support clinical diagnosis. Among the results obtained, the DeepBreastCancerNet network achieved 99.35% accuracy in classifying breast cancer via ultrasound. They also report the results of the ThyNet network for thyroid diagnosis, in which it achieves an Area Under the Receiver Operating Characteristic Curve (AUROC) of 0.922, higher than the 0.839 achieved by radiologists. This demonstrates the potential of these two architectures when trained for this function, freeing up time for professionals to focus on other approaches. Furthermore, deep learning addresses critical healthcare access barriers, such as the high cost and time consumption of traditional diagnostics coupled with specialist shortages, especially in resource-limited settings [16]. Developing cost-effective, accessible AI models deployable on edge devices has the potential to democratize advanced diagnostics.

### 3.1. Core Principles

Advanced pulmonary diagnostic systems are built upon deep learning (DL) architectures, which play a fundamental role due to their ability to process and analyze highly complex medical data.

#### 3.1.1. Key Architectures and Their Applications

Convolutional neural networks (CNNs) are essential for imaging applications due to their ability to process image information effectively. By using a kernel and techniques such as max pooling or average pooling, they can reduce the complexity of an image by transforming it into a vector and processing the information more efficiently. These networks have been widely used in all types of imaging and, in medicine, they represent a significant advancement for the classification, detection, and segmentation of various types of diseases, such as the detection or classification of lung cancer.

Following the same example of identification, in Samia Dardouri’s work [16], the researchers successfully trained a CNN model capable of identifying areas affected by lung diseases based on chest X-rays (CXR) and computed tomography (CT) scans. The model was trained using pre-trained models and achieved high performance in detecting and classifying multiple diseases, including pneumonia, COVID-19, and normal lung conditions. This model reported an accuracy of 96%, a recall of 95.33%, an F1-score of 95.66%, and a precision of 97.24% on a dataset consisting of 6400 images.

In Baldwin’s study [23], validation was performed using CNN-based artificial intelligence tools, referred to as the lung cancer prediction CNN (LCP-CNN), designed to predict the risk of malignancy in pulmonary nodules using computed tomography (CT) images. To conduct this study, 1397 lung nodules from 1187 patients at three different hospitals were analyzed and compared with traditional models such as the Brock University model. The results were positive, as the network achieved an AUC of 89.6%, which is higher than the 86.8% achieved by the traditional Brock model.

In the paper by Galic’ [17], the authors demonstrated that CNNs are among the most widely used architectures in medical image analysis, primarily for classification, detection/localization, and segmentation. The review highlights specific applications in the classification of pulmonary nodules on CT scans, the detection of breast tumors on mammograms, the diagnosis of diabetic retinopathy, and the classification of brain tumors and cardiovascular diseases. Furthermore, they highlight that CNN-based architectures, such as U-Net, DeepLab, and Mask R-CNN, have achieved state-of-the-art results in medical segmentation, including brain tumors, lung nodules, polyps, and blood vessels. However, the article concludes that their clinical use still faces significant limitations, particularly the need for large volumes of labeled data, the risk of overfitting, low interpretability, data bias, and high computational cost.

Transformers, characterized by their self-attention mechanisms, are highly effective in modeling long-distance dependencies and capturing global correlations within data. This capability makes them particularly suitable for integrating diverse data sources. An exemplary application is PneumoFusion-Net, a multimodal framework designed for pneumonia diagnosis. This system leverages a Swin Transformer-based dynamic attention mechanism to integrate CT images, clinical text, numerical laboratory test results, and radiology reports. This comprehensive integration of disparate data types has resulted in a high classification accuracy of 98.96% for distinguishing bacterial from viral pneumonia [11].

The Swin Transformer’s shifted window mechanism contributes significantly to its efficiency, reducing computational complexity while maintaining global interaction across modalities. This is a critical advantage when processing high-resolution CT data, where traditional full-attention mechanisms would be computationally prohibitive. Beyond classification, deep learning models, including CNNs, applied to low-dose chest CT scans have demonstrated strong performances in predicting pulmonary function test results, such as forced expiratory volume in the first second (FEV1) and forced vital capacity (FVC). These models achieved accuracies ranging from 85.9% to 90.2% for predicting high-risk respiratory groups [24] (Table 1).

Variational autoencoders (VAEs) are commonly used as large-scale, unlabeled, self-supervised models to extract features from images, such as CT scans. VAEs can be used for autonomous feature extraction in images, be it edges, tones, textures, etc. They offer a significant advantage due to their compatibility with data fusion strategies; they can be applied by combining deep learning features extracted by the VAE with radiomic features, for example, which substantially improves diagnostic results. For example, in the work by Zecheng Zhu [12], they presented a model that fuses both features, which achieved an AUC of 0.952, outperforming independent models based solely on deep learning features, which obtained an AUC of 0.844, and radiomic features, which achieved an AUC of 0.944. This performance increases even further when epidemiological questionnaire data is incorporated into a comprehensive model, resulting in an AUC of 0.971. This suggests that data fusion improves the performance of models in medical applications.

While individual deep learning architectures, such as CNNs, Transformers, and autoencoders, yield good results, the best-performing models today are typically associated with applications that fuse more than one type of data; that is, multimodal systems, as demonstrated by the PneumoFusion-Net model and the comprehensive COPD diagnostic framework, which significantly improve accuracy by incorporating multiple types of input data, such as images, diagnostic findings, and numerical laboratory results. The integration of multiple input sources generates diverse data and helps overcome the limitations of analyses based on single, modal models; in turn, this enables a more robust diagnosis by providing a more comprehensive view of the patient’s condition. It also strikes a balance between the generality and specificity of the domain being analyzed: models trained for generic use, such as Transformers with large, heavy architectures trained on 248 generic datasets of skin types or fruits, for example. They offer power but are limited, as the literature suggests, that a model specifically developed and designed for a particular purpose demonstrates superiority when refining the domain in question, combined with effective and clean data augmentation, as well as adaptive optimization techniques. This approach to designing the network for a specific task, if applied solely to chest X-rays using well-labeled and diagnosed data from a distinct domain, yields better results and a more effective application, given that it will be used exclusively for that type of input.

#### 3.1.2. Transfer Learning

It is important to note that there is a significant shortage of labeled medical datasets; before any training begins, one must bear in mind that it is difficult to find data with multiple inputs for multimodal models from scratch [25]. A method known as “transfer learning” is necessary, as it leverages models that have already been trained on a specific dataset and can continue to be trained with additional data acquired in the future, making them more comprehensive. ImageNet, for example, is a model with precedents that aid this work when retrained.

The multimodal deep learning (MDL) approach is a good example of this methodology. It employs a set of five different pre-trained deep learning models: VGG-16, VGG-19, ResNet, AlexNet, and GoogleNet, which are designed to classify multiple classes of chest X-ray images with high accuracy [26]. The strategy has the advantage that each model contributes its own strengths, addressing challenges such as feature diversity and data imbalance, which are common in medical image classification. For lung cancer classification, for example, an innovative approach is used to select regions of interest (RoI) based on intensity from public datasets such as LIDC-IDRI and CIA. This approach incorporates improvements and supervision based entirely on feedback through transfer learning, which demonstrates an accuracy improvement of 97.84% [18].

In order to reduce the impact of the limited number of datasets in the medical field, data augmentation is a key technique for mitigating this problem; thus, neural networks are able to access a larger volume of data during training [16]. Data augmentation artificially adds data to existing datasets to make them more robust, thereby preventing the network from experiencing issues such as overfitting or a lack of accuracy due to insufficient data [27]. Among these techniques, two are RandFlip (which involves randomly flipping CT volumes along an axis) and RandRotate (random rotation of CT volumes by 90 degrees) [25]; more advanced methods, such as adaptations of RandAugment for 3D training and techniques like Mixup, Cotout, and Cutmix, which are focused on image classification; and semantic data augmentation (SDA) methods, such as Bayesian Semantic Random Data Augmentation (BSDA), generate new data rather than directly modifying the original image. Furthermore, the BSDA method is efficient and computationally inexpensive, making it suitable for use with various types of neural networks, as well as for two- and three-dimensional datasets across different modalities [27].

The relative efficacy of these augmentation techniques, including the Cutmix method, which yields the largest gains for lung cancer binary prediction, is discussed in Section 3.2.2.

Deep transfer learning-based computational models have achieved promising results in classifying fifteen different pulmonary diseases using chest radiographs, reporting high specificity (97.92%), sensitivity (97.30%), precision (97.94%), and AUC (97.61%) [26]. Despite these advancements, challenges persist, including model dependence on specific training data, issues with generalization across diverse datasets, effective integration of clinical metadata, and ensuring model interpretability. The acquisition of high-quality labeled data remains resource-intensive, and biases embedded in training data can lead to skewed predictions, necessitating careful attention during dataset curation and model 297 development.

The fundamental challenge in applying deep learning to medical domains is the scarcity of large, meticulously labeled datasets [25]. Transfer learning and data augmentation are consistently presented as primary solutions to this problem. This highlights a direct relationship: limited data leads to issues such as overfitting and poor generalization, which are then mitigated by leveraging external knowledge or artificially expanding the dataset. These techniques are not merely optimizations but serve as enabling technologies for deep learning in data-constrained medical environments, allowing for the development of robust models even when comprehensive, domain-specific labeled data are scarce.

### 3.2. Medical Image Processing

Medical image processing is a critical component of deep learning applications in pulmonary diagnosis, with specific techniques tailored for X-ray and CT modalities.

#### 3.2.1. X-Ray and CT Techniques

Accurate detection and precise segmentation of images of lung nodules on CT scans are indispensable prerequisites for the early diagnosis and appropriate treatment of lung cancer [28]. Deep learning methods, including Fully Convolutional Networks (FCN), Mask R-CNN, and U-Net, are widely adopted for medical image segmentation due to their ability to learn rich local features [29]. The primary objective of lung image segmentation is to expedite the extraction of relevant lung areas, thereby enhancing diagnostic efficiency for medical professionals. Traditional methods often encounter difficulties with small or hidden pulmonary nodules, making deep learning algorithms essential for their reliable detection. A significant challenge in this domain is the labor-intensive and subjective nature of manual segmentation, which frequently leads to inter- and intra-rater discrepancies. Deep learning offers a robust solution to automate this laborious task, improving consistency and reducing human variability.

Deep learning-based image reconstruction and noise reduction methods (DLIR) are increasingly being deployed in routine CT practice. These advanced methods can significantly improve image quality and enhance lung nodule detection, particularly in ultra-low-dose chest CT scans (0.07–0.14 mSv), which are comparable in radiation exposure to a single chest radiograph [30]. DLIR has demonstrated its capability to reduce image noise and increase nodule detection rates when compared to traditional iterative reconstruction methods. A general framework for assessing image quality in DLIR involves projection-domain lesion and noise insertion, which allows for the simulation of various dose levels and anatomical variations, enabling comprehensive evaluation of the reconstruction algorithms. The application of DLIR is particularly advantageous for pediatric patients, who are more sensitive to radiation, as it can potentially reduce the need for repeated CT simulations, thereby minimizing radiation exposure [31].

Beyond nodule detection, deep learning models are employed for a wide range of applications, including the identification and segmentation of tuberculosis, detection of COVID-19, and general categorization of radiographs, contributing to enhanced diagnostic accuracy across various pulmonary conditions. Transfer learning approaches, utilizing pre- trained models such as MobileNetV2, VGG-16, and ResNet50V2, are applied to categorize various chest disorders using X-ray images, with the aim of improving the efficiency and accuracy of computer-aided diagnostic systems [32].

The interplay between image quality and diagnostic accuracy is a critical aspect of deep learning in medical imaging. The discussion highlights both image processing techniques, such as segmentation and noise reduction, and their direct impact on diagnostic outcomes like nodule detection and disease classification. Specifically, DLIR is shown to reduce image noise, which in turn increases nodule detection rates and improves measurement accuracy. This establishes a clear relationship: advancements in image quality, facilitated by DLIR, directly lead to enhanced diagnostic accuracy. This suggests that investment in DL-based image reconstruction and enhancement is as crucial as developing sophisticated classification algorithms, as the inherent quality of the input data fundamentally constrains the performance of subsequent diagnostic tasks.

Furthermore, the ability of DLIR to improve image quality and nodule detection for ultra-low-dose chest CTs is a significant advancement. This directly addresses the concern of radiation exposure, which is particularly relevant for large-scale screening programs and for vulnerable populations such as pediatric patients. The broader implication is that deep learning is not only refining diagnostic precision but also making screening procedures safer and more broadly applicable. This could transform public health strategies for lung cancer and other pulmonary conditions by mitigating a substantial barrier to repeated imaging, thereby enabling more frequent and less risky examinations for at-risk populations.

#### 3.2.2. Data Augmentation in Medical Domains

Data augmentation serves as a crucial regularization technique for deep neural networks, especially in medical imaging tasks where labeled datasets are often limited. It plays a vital role in addressing issues such as model bias, overfitting, and inaccuracies that arise from insufficient training data [27].

Various techniques are employed to augment medical image datasets. Common geometric transformations include RandFlip, which randomly flips CT volumes along a selected axis, and RandRotate, which randomly rotates CT volumes by 90 degrees [25]. More advanced methods, such as adaptations of RandAugment, have been developed for 3D inputs [25]. Techniques like Mixup, Cutout, and Cutmix, originally designed for general image classification, have been successfully adapted for 3D CT volumes in lung cancer prediction tasks.

A more sophisticated approach is semantic data augmentation (SDA), which generates new data at the feature level rather than directly modifying images. Bayesian random semantic data augmentation (BSDA) is a computationally efficient SDA method that can be seamlessly integrated into different neural networks architectures and applied to both 2D and 3D medical image datasets across multiple imaging modalities [27].

The effectiveness of data augmentation in improving model performance is highly dependent on the specific approach utilized. For instance, the Cutmix method demonstrated the highest average performance improvement across accuracy, F1 score, and AUC for lung cancer binary prediction. Specifically, it resulted in gains of 1.07% in accuracy, 3.29% in F1 score, and 1.19% in AUC [25]. Data augmentation can also enhance prediction performance by rebalancing training sets and incorporating moderately synthetic data. It is important to note that the efficacy of both online and offline data augmentation methods is highly sensitive to the prediction model, underscoring the necessity for careful selection of the optimal method for a given task. In certain scenarios, traditional data augmentation methods can provide more stable and higher performance compared to some state-of-the-art online approaches.

While the primary function of data augmentation is to address data scarcity, its impact on performance is highly dependent on the specific method employed. The observation that “certain traditional methods can provide more stable and higher performance compared to SOTA online data augmentation approaches” [25] indicates that data augmentation is not a universal solution, but a nuanced technique requiring careful selection and adaptation based on the model and dataset characteristics. This suggests that researchers should move beyond simply applying generic augmentation strategies and instead conduct rigorous comparative studies to identify the most effective approaches for particular medical imaging tasks. This precision in applying data augmentation transforms it from a mere volume enhancer into a critical tool for optimizing model performance.

The mention of the MED-DDPM data augmentation approach improving prediction performance by “rebalancing the training set and adding moderately synthetic data”, alongside discussions of semantic data augmentation operating at the feature level, illustrates a progression from simple geometric transformations to more sophisticated generative and semantic methods. This trend towards increasingly sophisticated synthetic data generation, if rigorously validated, holds the potential to significantly reduce the burden of manual annotation and data collection, thereby accelerating AI development in medicine. However, it also raises important questions regarding the generalizability and trustworthiness of models trained on synthetic data in real-world clinical scenarios, necessitating the development of robust validation frameworks to ensure their reliability and safety.

### 3.3. Acoustic Signal Processing

Acoustic signal processing for pulmonary diagnosis involves extracting meaningful features from lung sounds to aid in the detection and classification of respiratory diseases. The analysis of lung sounds extensively leverages spectral features such as Mel-frequency cepstral coefficients (MFCCs), spectrograms, and Mel spectrograms to capture the distinct sonic signatures of various respiratory conditions [33,34].

Mel spectrograms are particularly effective as their design is inspired by the human auditory system’s sensitivity to variations in lower frequencies and its logarithmic perception of loudness. The process involves transforming lung sound samples from the time domain to the frequency domain using the Short-Time Fourier Transform (STFT) and then mapping these frequencies to the Mel scale. This generates a two-dimensional image where columns represent time windows and rows represent frequencies on the Mel scale, with each value corresponding to the signal’s log amplitude for a specific frequency and time segment [33].

Continuous Wavelet Transform (CWT) scalograms offer another powerful approach for analyzing non-stationary audio signals. CWT preserves temporal resolution and decomposes signals into different frequency components using wavelets, enabling simultaneous analysis in both frequency and time domains. A complex Morlet wavelet is frequently employed as the mother wavelet due to its ability to capture coherence between harmonic frequencies and provide optimal time localization [33]. Comparative studies have evaluated the performance of models trained with spectrograms, Mel spectrograms, and MFCCs for abnormal symptom detection [35]. The Mel-spectrogram-based model consistently achieves the highest classification accuracy at 97.13%, outperforming models based on spectrograms (95.22%) and MFCCs (93.78%). This superior performance is attributed to the Mel spectrogram’s ability to emphasize critical acoustic features through Mel-filter banks, which better reflects human auditory perception and effectively retains essential characteristics while reducing data dimensionality.

The TriSpectraKAN model further demonstrates the benefits of a multimodal approach by combining MFCCs, chromagrams, and Mel spectrograms through a hybrid network for COPD detection [13]. This integration of multiple audio features captures a broader range of lung sound characteristics, leading to improved diagnosis accuracy compared to methods relying on single feature types (Table 2).

The superior performance of Mel spectrograms is directly linked to their design, which is inspired by the human auditory system’s sensitivity. This indicates that features engineered to mimic human perception, particularly for auditory signals, lead to more effective representations for machine learning models. This highlights the value of interdisciplinary approaches, where insights from psychoacoustics can significantly enhance feature engineering for medical audio signals, leading to substantial performance gains in diagnostic accuracy.

While Mel spectrograms perform exceptionally well individually, the TriSpectraKAN model demonstrates that combining MFCCs, chromagrams, and Mel spectrograms through a hybrid network further enhances COPD diagnosis. This illustrates that even if one feature type is individually superior, a multimodal fusion of different spectral features can provide a more comprehensive and robust analysis. This approach captures unique sonic signatures that might be missed by relying on a single feature type, thereby reinforcing the broader trend of multimodal data fusion observed in image processing and extending its benefits to acoustic signals for improved diagnostic precision and robustness.

#### 3.3.1. Preprocessing: Noise Filtering and Segmentation

Preprocessing acoustic signals is crucial for accurate pulmonary diagnosis, as raw lung sound auscultation is often challenging and unreliable due to inherent noise and interference. This is particularly true for neonatal chest sounds, where noise frequently overlaps with heart and lung sounds in both time and frequency domains. The poor signal-to-noise ratio in thoracic lung sounds is a significant factor contributing to diagnostic inaccuracies.

To address noise contamination, the Single-channel Blind Source Separation (SCBSS) framework has been proposed to separate newborns’ lung and heart sounds from noisy chest sound recordings [36]. This method begins by decomposing the signal into a multi- resolution representation using time–frequency transforms, such as Stationary Wavelet Transform (SWT) or Continuous Wavelet Transform (CWT). Subsequently, source separation algorithms like Principal Component Analysis (PCA), Periodic Component Analysis (πCA), and Second Order Blind Identification (SOBI) are applied to isolate the desired components. SCBSS methods have been shown to yield higher-quality heart and lung sounds and more reliable heart and respiratory rate estimations compared to raw signals or conventional fixed band-pass filtering. Beyond these techniques, deep learning-based audio enhancement preprocessing steps can significantly improve the robustness and clinical applicability of automatic respiratory sound classification systems, especially in noisy real-world conditions.

Preprocessing of lung sounds frequently involves segmenting recordings into individual respiratory cycles, which can vary considerably in duration, ranging from 0.2 s to 16 s with an average of 2.7 s. To ensure a consistent input size for deep learning models, a padding technique is employed, modifying each cycle to a fixed duration, typically six seconds, using zero padding. This standardization is critical because adaptive average pooling performs less effectively compared to models trained on fixed-size signals. Following these preprocessing steps, respiratory cycles are often transformed into time–frequency scalograms using parallel techniques like CWT and Mel Spectrograms, providing distinct representations for subsequent analysis.

The presence of noise and interference is a significant impediment to accurate lung sound diagnosis. The SCBSS framework and general audio enhancement preprocessing are presented as effective solutions to this problem. This establishes a clear relationship: high noise levels degrade diagnostic accuracy, while effective preprocessing techniques significantly improve it. This highlights that sophisticated preprocessing is not merely an optional step, but a fundamental requirement for developing robust and clinically applicable AI models for acoustic pulmonary diagnosis, particularly in uncontrolled, real-world environments where noise is ubiquitous.

Signal processing thus acts as a pre-emptive quality control mechanism: it actively improves raw data quality before it is fed into deep learning models. This is distinct from image processing, where noise reduction is typically part of reconstruction, or post-world environments, where processing is a fundamental prerequisite for initial feature extraction [37]. The necessity for segmentation and padding to create consistent input lengths for deep learning models, despite the inherent variability of respiratory cycle durations, underscores a practical challenge in data preparation. The rationale provided is that adaptive average pooling performs poorly when compared to fixed-size signals. This suggests that even powerful deep learning models benefit substantially from standardized, pre-conditioned inputs. The implication is that effective data preparation, even through seemingly simple techniques like padding, is crucial for achieving model stability, faster convergence, and improved generalization across diverse patient data, all of which are vital for successful clinical deployment. This emphasizes that meticulous attention to data preprocessing is as important as the complexity of the deep learning architecture itself.

#### 3.3.2. Temporal Models: LSTM, GRU, and 1D Transformers

Temporal deep learning models are crucial for analyzing the sequential nature of acoustic signals in pulmonary diagnoses, capturing time-dependent patterns in lung sounds.

Recurrent neural networks (RNNs), specifically Long Short-Term Memory (LSTM) and Gated Recurrent Unit (GRU) networks, are highly effective for time-series analysis, and are widely applied in lung sound analysis for tasks such as breath phase detection and adventitious sound detection [33]. Hybrid deep learning architectures often combine convolutional neural networks (CNNs) for feature extraction and dimensionality reduction with LSTMs to identify and retain temporal dependencies within the data [38]. Performance comparisons of various RNN variants have been conducted [39]:

• **GRU versus LSTM:** GRU-based models generally outperformed LSTM-based models in most tasks, exhibiting F1 scores that were 0.7% to 9.5% higher when a CNN was not included. They also demonstrated superior Area Under the Curve (AUC) values in general, suggesting that GRU models can be computationally more efficient.
• **Unidirectional versus Bidirectional:** Bidirectional models (BiLSTM, BiGRU) consistently surpassed their unidirectional counterparts across all tasks, with F1 score improvements ranging from 0.4% to 9.8%. This highlights the effectiveness of processing information in both forward and backward temporal directions to capture more comprehensive context.
• **With versus Without CNN:** Models incorporating a CNN generally outperformed those without a CNN in 26 out of 32 compared pairs [39]. This improvement was particularly notable in continuous adventitious sound (CAS) detection tasks, where F1 scores improved by 26.0% to 30.3%, and AUC values increased by 0.067 to 0.089 [39]. This suggests that CNNs are effective at extracting local positional arrangement information that is crucial for CAS detection.

Transformer models, including the transformer–CP (circular positional encoding) model, are increasingly being explored for lung sound classification. These models incorporate position information into input sequences using circular positional encoding to effectively handle the cyclic nature of sound data. Audio Spectrogram Transformers (ASTs) leverage pre-trained audio models and a Patch-Mix strategy to enhance performance and prevent overfitting in respiratory sound classification.

The advantages of ASTs are notable in noisy respiratory sound settings. On the ICBHI dataset, audio enhancement preprocessing improved a fine-tuned AST by 11.71 percentage points in sensitivity and 1.40 points in the ICBHI score; when combined with the Patch-Mix AST strategy, the corresponding improvements were 17.08 percentage points in sensitivity and 1.60 points in the ICBHI score [40]. Furthermore, Transformer-based feature encoders can be pre-trained on unlabeled data through self-supervised learning, and a random masking mechanism can further enhance their performance [34] (Table 3).

The comparative analysis of temporal models reveals the complementary strengths of CNNs and RNNs/Transformers. While RNNs like LSTM and GRU are adept at handling temporal dependencies, the integration of CNNs significantly enhances accuracy, particularly for detecting continuous adventitious sounds. This indicates that CNNs excel at extracting local, spatial features from spectrograms (treating them as images), while RNNs and Transformers are superior at capturing the sequential and temporal relationships of these features over time. The implication is that hybrid models, which combine the strengths of both convolutional and recurrent/attention mechanisms, are likely to be the most effective for complex audio classification tasks in pulmonary diagnosis, offering a more comprehensive understanding of the acoustic patterns.

Despite the strong performance of LSTM and GRU models, the emergence of Transformer models in lung sound classification signifies a notable shift in architectural preference. Transformers, with their self-attention mechanisms, are highlighted for their ability to capture long-range dependencies and process sequences in parallel. This capability potentially overcomes some limitations of traditional RNNs in handling very long sequences or achieving computational efficiency. The broader implication is that attention-based architectures, which have revolutionized fields like Natural Language Processing and computer 560 vision, are increasingly becoming the state of the art for temporal modeling in medical audio. This promises more robust, scalable, and potentially more accurate solutions for analyzing complex respiratory sound patterns, leading to more reliable diagnostic tools.

#### 3.3.3. Representation Learning for Acoustic Signals

Representation learning, particularly through self-supervised learning (SSL), is emerging as a powerful solution for medical audio analysis, directly addressing the significant challenge posed by the need for meticulously annotated data. SSL capitalizes on vast amounts of unlabeled data by devising proxy tasks that allow models to learn meaningful representations directly from the data itself, without requiring human annotation.

Wav2Vec 2.0 stands out as a prominent SSL model designed to learn representations directly from raw audio waveforms. It comprises two main components: a convolutional feature encoder that converts raw audio into latent representations, and a Transformer-based context network that processes these into higher-level context vectors. The model’s pretext task involves a contrastive learning approach in which masked latent representations are predicted from a set of candidates. In medical audio applications, Wav2Vec 2.0 and related SSL models are therefore better interpreted as representation-learning backbones whose performance depends on the downstream task, dataset, and fine-tuning protocol, rather than as models associated with a single general accuracy range [41].

While not always explicitly named, the underlying principles of Wav2Vec and HuBERT are implicitly integrated into many Transformer-based approaches for medical audio. For instance, the Audio Spectrogram Transformer (AST) bypasses extensive preprocessing by leveraging self-attention mechanisms to preserve diagnostic features directly from spectrograms. This aligns with the core objective of Wav2Vec and HuBERT: to learn rich representations directly from raw or minimally preprocessed audio [42]. These models learn meaningful features directly from the data, reducing reliance on labor-intensive, handcrafted feature engineering. The self-attention mechanism, central to these models, enables them to learn contextualized representations by understanding complex relationships between different parts of the audio sequence [42].

A fundamental concept underpinning Wav2Vec and HuBERT is transfer learning, which involves utilizing pre-trained models and fine-tuning them for specific downstream tasks [18]. This allows models to learn general audio representations from vast unlabeled datasets, which can then be effectively transferred and adapted to smaller, labeled medical datasets for specific diagnostic applications.

SSL models offer a promising pathway for developing automated and objective lung sound analysis systems, significantly enhancing the accuracy and reliability of respiratory disease diagnosis. This is particularly beneficial in remote areas lacking experienced medical professionals or during public health crises, where large-scale respiratory screenings are needed. By eliminating the need for extensive manual feature engineering, SSL models can capture intricate patterns directly from raw audio, leading to the development of scalable and efficient diagnostic tools that can be widely deployed.

The primary bottleneck for applying deep learning in medicine is the scarcity of large, labeled datasets. Self-supervised learning models, such as Wav2Vec and HuBERT, are specifically designed to learn from unlabeled data. This directly addresses the data scarcity problem by leveraging the vast amounts of readily available unlabeled medical audio. This approach removes the dependency on costly human annotation, thereby enabling the training of much larger and more complex models. This, in turn, leads to greater scalability and efficiency in developing diagnostic tools, as the constraint of manual labeling is significantly reduced.

The pre-training of Wav2Vec 2.0 on large amounts of unlabeled audio and its subsequent adaptation to downstream medical audio tasks points towards the concept of “foundation models” in medical audio [41,43]. These are large, pre-trained models that capture general audio representations, which can then be adapted to specific clinical classification tasks. This represents a shift from developing task-specific models from scratch to leveraging powerful, pre-trained general audio models. This approach could significantly accelerate research and development, reduce computational costs, and lead to more robust and generalizable AI diagnostics in pulmonary medicine, mirroring the transformative impact observed in Natural Language Processing with models like BERT and GPT.

## 4. Databases and Data Management

In clinical practice, it is difficult to obtain X-rays from patients with rare diseases. This is known as class imbalance [44]. Therefore, the detection of rare critical diseases is complex, since machine learning models are primarily trained on data from common cases [45]. The true constraint is not raw data volume but the capacity to transform heterogeneous clinical information into high-quality, ethically sourced, and precisely annotated datasets. Addressing this requires investment in advanced data curation pipelines, privacy-preserving technologies [46], and cross-institutional collaboration to ensure AI generalizability and clinical utility.

Another difficulty with multilabel classification problems, such as ChestX-ray14, arises when patients suffer from more than one disease, which complicates the training process. To address this, researchers employ various strategies, such as specialized loss functions like Focal ZLPR (FZLPR) or Focal Loss, which help manage class imbalance [47]. They also apply dynamic resampling techniques to achieve a more balanced representation of minority classes during training [48], in addition to various data augmentation methods.

Artificial intelligence systems can adopt the biases present in the data used to train them. This can lead to systematic errors and inconsistent results when applied to different demographic groups. As a result, these flaws often manifest as underdiagnosis among vulnerable or marginalized populations, disproportionately affecting certain subgroups based on their ethnicity, gender, or age [14]. These differences have several causes. Among the main ones are a lack of demographic diversity in the original databases, differences between institutions in imaging protocols and equipment, and biases on the part of specialists when manually labeling and annotating the data [49]. Assessing and mitigating these biases is part of the technical challenges of the study. This is because there are numerous medical image databases that lack demographic information. Added to this is the inconsistency in the categorization of population data and the lack of a standardized, universally accepted statistical consensus on the definition and measurement of bias [50].

Class imbalance and population biases in medical datasets cause not only technical problems but also ethical ones, since artificial intelligence models trained on under-representative data tend to exacerbate pre-existing health and social inequalities. Failure to understand these biases leads to poor diagnosis, disproportionately affecting vulnerable demographic groups. Consequently, a paradigm shift is being promoted in the development and evaluation of these systems, moving from an approach focused exclusively on technical performance toward one that integrates equity metrics and requires transparency regarding the demographic composition of the data used.

That is why various mitigation strategies have been proposed and studied. These include preprocessing methods, such as resampling or dataset augmentation, which aim to balance the data before model training. Processing methods incorporate adversarial components into the base model during training or give greater weight to specific loss functions during training, thereby reducing bias. Postprocessing techniques introduce perturbations into the input images after the model prediction. It is important to note that simply oversampling minority groups is not sufficient, since systematic adjustments are often required to achieve equitable performance [15,51]. Furthermore, research indicates that technical parameters related to image acquisition can influence the ability of AI models to predict patient demographics, and actively addressing these factors can significantly reduce bias [52]. This means that bias mitigation is not a one-off solution, but a continuous and multifaceted process that must be integrated at every stage: from data collection and model training to post-deployment monitoring. This approach requires interdisciplinary collaboration among AI developers, clinicians, ethicists, and policymakers to ensure that AI tools are not only accurate, but also equitable and reliable in real-world clinical applications (Table 4).

### Public Acoustic Datasets

Public acoustic datasets underpin most of the work on AI-based diagnoses from lung sounds, and their characteristics shape what kinds of models can be trained and tested. The International Conference on Biomedical and Health Informatics (ICBHI) 2017 Respiratory Sound Database remains the reference benchmark in this area [33]. The database contains 920 audio recordings collected from 126 different patients, comprising 5.5 h of breathing cycles that have been reviewed and labeled by medical professionals [54]. Since different equipment was used, the sampling frequency of the original audio files ranged from 4 kHz to 44.1 kHz. To avoid confusion in the algorithms, the higher-quality audio files were downsampled to 4 kHz. The breathing audio clips were labeled as adventitious sounds, that is, abnormal breathing noises, since they are heard when strange sounds occur while inhaling: either as a wet crackling sound (which happens when there is fluid in the lungs) or as a wheezing sound (typical of asthmatics when their bronchial tubes constrict). They also indicate if the patient is unlucky enough to have both sounds together. The 126 patients in this database suffer from a variety of respiratory diseases. These include: pneumonia, lower respiratory tract infection (LRTI), asthma, bronchiectasis, upper respiratory tract infection (URTI), bronchiolitis, and chronic obstructive pulmonary disease (COPD).

The Coswara dataset is a crowdsourced collection assembled between April 2020 and February 2022. It contains respiratory recordings and detailed metadata from 2635 individuals, divided into SARS-CoV-2 negative (1819), positive (674), and recovered (142) participants [55]. Nine sound categories were captured per subject, covering breathing, coughing and speech, together with metadata on age, gender, geographic location, self- reported symptoms, pre-existing respiratory conditions, and COVID-19 test status. The geographic distribution is uneven: most participants are based in India, which limits how directly the dataset generalizes to other populations. The full 65 h of audio were manually annotated for recording quality, and the original authors also conducted a subgroup analysis examining how gender, location, recording date, and language proficiency affected COVID-19 detection performance [55].

The AudioCOVID dataset is part of a broader wave of acoustic resources assembled during the pandemic to explore whether COVID-19 can be detected from speech, cough, or breathing sounds [56]. Detailed specifications for this dataset are limited in the published literature, and direct comparisons with ICBHI or Coswara should be made with caution. A common practice in this area has been to aggregate multiple sources (the CoronaHack respiratory sound dataset is a frequently used component) to compensate for the small number of confirmed COVID-positive recordings available from any single collection [57]. The classification targets in these aggregated corpora vary, but typically span healthy controls, pneumonia, COPD, and a residual category covering COVID-19, asthma, LRTI, URTI, bronchiolitis, and bronchiectasis.

HF_Lung_V1 is an open access lung sound database that was subsequently expanded into HF_Lung_V2, with a 1.43-fold increase in the number of audio files [58]. It has been used extensively to benchmark deep learning models for respiratory sound analysis, including hybrid architectures such as convolutional neural network–bidirectional gated recurrent unit (CNN-BiGRU) models, on tasks that include inhalation and exhalation detection, as well as continuous and discontinuous adventitious sound (CAS, DAS) recognition [39]. Two recurring difficulties have shaped the work on this dataset: label noise and overlapping sound events within the same recording. Table 5 summarizes the four datasets discussed in this section in terms of duration, sound types, target diseases, and main limitations.

#### Challenges in Environment and Variability

One of the main difficulties in acoustic pulmonary diagnosis is environmental noise, which overlaps with diagnostically relevant heart and lung sounds in both the time and frequency domains [59]. The noise-isolation strategies available to researchers—classical SCBSS (SWT/CWT decomposition followed by PCA, πCA, or SOBI) and deep learning-based audio enhancement modules—are described in detail in Section 3.3.1. In the dataset context, the key implication is that noise characteristics are not uniform across collections: the ICBHI database, for instance, was recorded with heterogeneous equipment at sampling rates ranging from 4 kHz to 44.1 kHz, while HF_Lung_V1/V2 exhibits overlapping adventitious events and noisy labels [39]. These dataset-specific artifacts mean that a model trained on one collection may not transfer cleanly to another, even when the same preprocessing pipeline is applied [40].

The combination of the previously described technical and physiological variables justifies the suboptimal diagnostic performance of traditional acoustic auscultation. The factors influencing diagnosis are lung physiology, as they depend on airflow, lung, and residual volume, body posture, the presence of secretions, and background noise produced by heartbeats and muscle contractions [34]. Lung sounds are variable. Furthermore, the terminology used to describe lung sounds is not universal, meaning that the same acoustic event can be labeled differently depending on who records it [6]. In addition, recording conditions and the specific equipment used, such as different stethoscope or microphone models, introduce their own variability into the resulting datasets [60]. The response from the deep learning community has been twofold. On the modeling side, architectures designed to tolerate variability, particularly convolutional neural networks (CNNs) and recurrent neural networks (RNNs), are now the default. Regarding the data, preprocessing steps, such as padding respiratory cycles to a fixed length, normalize the input dimensions and help models generalize more reliably across recordings.

Working with acoustic data requires balancing fidelity and generalizability. Lung sounds vary between patients, recording conditions, and equipment, and the standard response has been to compress this variability through preprocessing (resampling, padding, filtering) and simulate greater variability by augmenting the data (noise injection, temporal stretching, pitch shifting) [61]. Both strategies help generalize models, but neither comes without a cost. Aggressive filtering or excessive standardization can eliminate the acoustic details that truly distinguish one pathology from another [62], and since the frequency band containing diagnostic information varies between patients, a fixed filter chosen for the average patient will inevitably discard useful information for some. The question, then, is not whether to preprocess, but how much to preprocess before the gain in generalizability is offset by the loss of diagnostic content. Two more recent strategies avoid the dilemma rather than solve it directly: self-supervised models trained on raw audio [41], and multimodal fusion approaches [13], where both absorb variability through representation learning rather than removing it through filtering.

## 5. Discussion

### 5.1. Synthesis of Main Findings

Three patterns stand out across the studies we reviewed. CNNs are still the workhorse for chest X-ray and CT classification, with reported accuracies sitting in the 92–97% band on multi-disease benchmarks (Table 1); though, as we noted earlier, those numbers come almost entirely from a handful of Kaggle splits and tend to drop on external cohorts. Transformer encoders are catching up: Swin Transformers in imaging and Audio Spectrogram Transformers in acoustics now match or beat CNN baselines, and the gap widens further when they are pre-trained in a self-supervised fashion. The clearest gains, however, are not architectural at all. They come from fusing modalities. Pairing CT with genomic data, auscultation with spirometry, or imaging with EHR text repeatedly yields double-digit improvements over the strongest unimodal baseline, and this pattern is robust enough that we would expect it to generalize to new pathologies as paired datasets become available.

### 5.2. Radiological Challenges and the Benchmark-to-Clinic Gap

The accuracy figures reported in Table 1 and Table 2 and throughout Section 3 and Section 4 should not be interpreted in isolation from their methodological limitations. As shown in Table 1, CNN and ViT architectures yield classification accuracies spanning 92–97%; nevertheless, such figures are obtained exclusively from Kaggle-sourced chest X-ray repositories or single-center CT datasets. Similarly, the acoustic performance metrics in Table 2 and Table 3 (including the 97.13% Mel-spectrogram accuracy and AST sensitivity improvements of up to 17.08 percentage points) remain confined to evaluation on the ICBHI 2017 benchmark (126 patients, heterogeneous recording equipment). Of particular methodological significance is the fact that benchmark-level performance, however strong, cannot be equated with validation on independent hospital cohorts; the two differ in terms of population diversity, acquisition variability, and clinical representativeness.

Image appearance in CT is jointly governed by scanner model, acquisition protocol, reconstruction kernel, slice thickness, and patient positioning; classifiers trained within a single institutional setting consistently exhibit AUC degradation of several percentage points (and frequently far greater losses) when deployed across different scanners [30]. Notably, both ChestX-ray14 and CheXpert were compiled within single academic institutions in the United States, resulting in demographic distributions that are neither balanced nor representative of the broader global patient population [49,53]. The generalizability of models trained on such resources is further constrained by demographic and technical heterogeneity; when applied to institutions with differing equipment or patient populations, performance decreases are both predictable and well-documented in the radiology AI literature [50].

Annotation quality represents a related but distinct methodological concern. Radiological labels in large-scale datasets are frequently derived from automated Natural Language Processing of radiology report text rather than structured expert consensus review, introducing label noise that varies systematically with reporting style and institutional convention [28]. Inter-reader variability remains considerable even among experienced radiologists when interpreting subtle findings such as ground-glass opacities or early-stage nodules; a model trained to agree with one reader may systematically disagree with another, and such discordance reflects the inherent ambiguity of the reference standard itself rather than model failure. In the absence of structured inter-annotator agreement metrics reported alongside performance figures, the extent to which algorithmic performance can be meaningfully improved remains difficult to establish.

These observations do not preclude the use of benchmark figures as relative comparisons across methods; they do, however, caution against interpreting such figures as absolute estimates of clinical utility. Confidence intervals are seldom reported, external validation results remain exceptional rather than standard practice, and dataset overlap between training and evaluation partitions is rarely verified explicitly. Where prospective clinical trials of imaging AI systems have been conducted, the observed effect sizes have consistently been smaller than those suggested by retrospective benchmark evaluations [14]. Integrating this interpretive caveat throughout the synthesis (rather than confining it to the limitations section) affords readers a more accurate assessment of the current state of the field.

Four methodological dimensions warrant explicit attention across the studies reviewed here. First, dataset quality: as documented in Section 4, label noise arising from automated NLP annotation of radiology reports, inter-reader variability, single-institution sampling, and demographic imbalance in widely used benchmarks such as ChestX-ray14 and CheXpert limit the reliability of the reported figures. Second, external validation remains the exception rather than the rule: as shown in Table 6 (Section 5.3), only 5 of the 18 key studies assessed report any form of cross-dataset or multi-center evaluation. Third, reproducibility is constrained by the fact that dataset overlap between training and evaluation partitions is rarely verified explicitly, and no pre-registered protocol underpins this review. Fourth, risk of overfitting is a persistent concern: small or single-source datasets coupled with the absence of independent test sets are the primary driver of inflated benchmark figures, and data augmentation and self-supervised pre-training are the main mitigation strategies identified in the reviewed literature.

### 5.3. Methodological Quality Assessment of Included Studies

To respond to the reviewers’ request for a more structured assessment of methodological quality, Table 6 summarizes, for the key studies discussed in Section 3 and Section 4, five indicators that bear directly on the reliability of the reported performance figures: whether the dataset used is public or private (and its approximate size), whether any form of external validation was performed, the headline performance metric as reported by the original authors, and whether the study was peer-reviewed or remains a preprint. The reported metrics are included for reference only and must not be interpreted as a ranking: they span incomparable tasks (COPD detection, COVID-19 screening, nodule malignancy, multilabel disease classification, image reconstruction quality, adventitious sound detection), different disease categories, and heterogeneous evaluation protocols, so any cross-row numerical comparison would be methodologically unsound.

**Table 6:** Methodological quality indicators for key studies discussed in this review.

Ref.	Study	Dataset (Public/Private; Approx. Size)	External Validation	Reported Performance (Metric)	Pub. Type
[16]	Dardouri (2025)	Public (Kaggle CXR; 6400 images)	No (internal split)	97.24% accuracy; 95.33% recall (CXR 3-class)	Peer-reviewed
[23]	Baldwin (2020), LCP-CNN	Private (3 UK hospitals; 1397 nodules, 1187 patients)	Yes (multi-center)	AUC 89.6% (nodule malignancy)	Peer-reviewed
[11]	Wang (2025), PneumoFusion-Net	Public (CT + text + labs; n not specified)	No (5-fold CV)	98.96% accuracy (multimodal pneumonia)	Peer-reviewed
[12]	Zhu et al. (2024)	Public (CT; 2983 participants)	No (single-site)	AUC 0.971 (COPD fusion model)	Peer-reviewed
[18]	Ahmed et al. (2025)	Public (LIDC-IDRI/CIA; n not specified)	No	Not extracted	Preprint (medRxiv)
[26]	Kumar (2025), MDL	Public (2 CXR datasets; n not specified)	Yes (cross-dataset)	AUC 97.61%; specificity 97.92% (15 diseases)	Peer-reviewed
[30]	Jiang (2022), DLIR	Public (203 participants; ultra-low-dose CT)	No (prospective, single-site)	Detection rate 75.8% DLIRvs 73.3% ASIR-V †	Peer-reviewed
[33]	Khan et al. (2024)	Public (ICBHI 2017; 126 subjects)	No (internal split)	94.16% acc. (8-class); 79.61% (4-class); 85.61% (binary)	Peer-reviewed
[13]	Roy (2025), TriSpectraKAN	Public (ICBHI + KAU + RD@TR; multi-dataset)	Yes (cross-dataset)	95.22% accuracy; F1 97.8% (COPD detection)	Peer-reviewed
[36]	Fattahi (2022), SCBSS	Private (neonatal chest sounds)	No	Not extracted	Peer-reviewed
[39]	Hsu et al. (2021)	Public (HF_Lung_V1; 9765 audio files)	No (internal split)	Not extracted (benchmark study, multiple RNN variants)	Peer-reviewed
[20]	Tabik (2020), COVIDGR	Public (COVIDGR-1.0; 852 images)	No (25 repeated runs, internal)	Sensitivity 72.6%, accuracy 76.2% (COVID-19 CXR) ‡	Peer-reviewed
[21]	Chowdhury (2020)	Public (COVID-19 Radiography DB; 13,808 images)	No (8:1:1 split)	98.3% accuracy (COVID vs. pneumonia vs. normal)	Peer-reviewed
[55]	Bhattacharya (2023), Coswara	Public (2635 subjects, India)	Limited (demo-graphic/geographic subgroups)	AUC 91.5% sensitivity 64.6% at 95% specificity	Peer-reviewed
[58]	Hsu et al. (2022), HF_Lung_V2	Public (HF_Lung_V2; 303 subjects, 4138 recordings)	No (internal split)	Not extracted	Peer-reviewed
[40]	Tzeng et al. (2025)	Public (ICBHI 2017+ Formosa Archive)	Yes (cross-dataset)	+21.88% ICBHI score improvement (relative, audio enhancement)	Peer-reviewed
[63]	Strick et al. (2025)	Public (ChestX-ray14; ~112,120 images)	No (internal split)	AUC-ROC 0.85; F1 0.39 (14- disease multilabel)	Preprint (arXiv)
[19]	Riquelme & Akhloufi (2020)	Public (LIDC-IDRI; n not specified)	No	Not extracted	Peer-reviewed

† Detection rate comparison between reconstruction algorithms; not a classification accuracy metric. ‡ Conservative estimate from 25-run internal evaluation; severity-stratified results vary substantially.

Two patterns emerge from the table. First, external or cross-dataset validation remains the exception rather than the rule: of the eighteen studies, only five report testing beyond a single internal split, the multi-center LCP-CNN evaluation [23], the cross-dataset evaluations of [13,26,40], and the demographic/geographic subgroup analysis on the Coswara dataset [55]. The remaining studies, including those reporting the highest headline accuracy figures (e.g., PneumoFusion-Net [11] at 98.96% and Chowdhury et al. [21] at 98.3%), rely exclusively on within-dataset evaluation, which limits the generalizability of those figures. Second, a majority rely on public, single-source benchmarks, which facilitates reproducibility but also means reported metrics largely reflect within-dataset performance.

Regarding within-group comparability of the reported metrics, the studies naturally cluster into three groups that admit limited internal comparison. The imaging group [16,20,21,26,63] all report classification accuracy or AUC on chest X-ray images, but across different disease targets (pneumonia, COVID-19, and fifteen simultaneous pulmonary pathologies), making direct comparison of the absolute values uninformative. The lung sound group [13,33,39,40,58] share the ICBHI 2017 benchmark as a common reference and are therefore the most mutually comparable subset in the table; even here, however, differences in the number of output classes (binary, four-class, or eight-class) and in train/test split conventions prevent straightforward ranking. Studies targeting lung nodule characterization ([18,19,23]) use AUC as the primary metric but differ in clinical task (malignancy prediction vs. nodule type classification), dataset origin, and case mix. Finally, ref. [30] (Jiang et al. 2022) is not a classification study at all but an image reconstruction algorithm evaluated by nodule detection rate, a metric that is incommensurable with any other entry in the table. These structural differences reinforce the interpretive caution argued for above and are revisited in Section 5.5.

### 5.4. Practical Barriers to Clinical Implementation

Moving from a published model to a tool that runs in a hospital workflow involves a set of challenges that the technical literature tends to understate. On the data side, medical records in most hospitals are stored across multiple systems that do not share a common data model: imaging archives (PACS), laboratory information systems, electronic health records, and audio capture devices each have their own schema, and mapping between them requires sustained engineering effort. The HL7/FHIR interoperability standards provide a framework for this, but compliance is uneven and many legacy systems predate those standards [17]. Missing data handling is also non-trivial: patients who arrive in acute settings may have imaging but no spirometry, or vice versa, and multimodal models trained on complete observations can fail silently when inputs are absent at inference time. Few of the papers reviewed here describe a principled strategy for this scenario.

Regulatory pathways represent another friction point. Medical AI software is classified as a medical device in most jurisdictions, which means it requires premarket clearance before clinical use. The evaluation criteria used by regulatory bodies, typically based on prospective studies, predefined performance thresholds, and a defined intended-use population, are stricter than the retrospective benchmark evaluations that dominate this literature. The gap between what is required for publication and what is required for clearance is wide. Some of the most widely cited models in this review have not been submitted for regulatory review, let alone approved, and it would be misleading to describe them as clinically deployable.

Finally, workflow integration matters in ways that purely technical evaluations miss. A tool that increases diagnostic accuracy by five percentage points but adds six minutes to a radiologist’s reading time may not improve clinical outcomes if it creates a bottleneck elsewhere in the care pathway. Latency constraints in emergency settings, alert fatigue from high false-positive rates, and trust calibration, knowing when to follow and when to override the model’s recommendation, are active research areas but are rarely discussed in the same papers that report benchmark accuracy. Addressing these questions will require collaboration between technical developers and clinical teams that extends well beyond the publication of a new architecture. Earlier reviews [25,34] have covered much of the same ground, but almost always through a single lens either one modality (imaging or audio) or one disease (typically COVID-19 or lung cancer). A more detailed comparison makes the distinction concrete. Galic’ et al. [17] provided a broad survey of CNN architectures in medical imaging, but their scope encompassed breast cancer, diabetic retinopathy, brain tumors, and cardiovascular disease alongside pulmonary conditions; respiratory acoustics and EHR modalities were absent, and the cross-modal fusion question was never posed. Ahmed et al. [18] addressed the specific problem of data augmentation for 3D CT-based lung cancer prediction: valuable methodologically, but limited to a single imaging modality and a single disease. Riquelme and Akhloufi [19] focused on Transformer encoders for adventitious sound classification, an important contribution to the acoustic branch of this field, but one that does not engage with imaging or structured clinical data. Tabik et al. [20] and Chowdhury et al. [21] both examined COVID-19 detection from chest X-rays; each is restricted to one modality and one disease episode, and neither considers how imaging findings could be combined with acoustic signatures or electronic health records. What distinguishes the present work is that it holds the imaging, acoustic, and EHR literature side by side within a single timeframe (2019–2024) and lets the asymmetries between them become visible: differences in dataset size, label conventions, the metrics authors choose to report, and the maturity of the translational pipeline. That cross-modality juxtaposition surfaces a pattern a single- modality review cannot easily see. Acoustic deep learning is no longer bottlenecked by architecture; it is bottlenecked by the quality and standardization of the datasets it has to learn from a situation analogous to where imaging AI was roughly five years earlier. Imaging has moved to a different set of problems. The hard questions there are no longer about accuracy on a held-out test set; they are about whether the model still works on a hospital it was not trained on, whether it works equally well across demographic groups, and whether the accuracy gain translates into a clinical outcome gain. By tracing both studies simultaneously, we can show how these transitions unfold and where the acoustic community is likely to encounter the same obstacles the imaging community is now working through.

No previous review in this period simultaneously addresses all three modalities: imaging, respiratory acoustics, and EHR data across the full spectrum of major pulmonary pathologies within a bounded and current timeframe (2019–2024). The works cited above each occupy a well-defined but narrower slice of this space: either they are multi-disease but single-modality [17,34], or they are multi-modality in principle but restricted to a single-disease episode [20,21], or they address a specific technical challenge (data augmentation) rather than synthesizing the state of the art [25]. The present review is, to our knowledge, the first to hold these three modalities side by side across four major respiratory diseases within this timeframe, and to examine both the technical and translational dimensions of their integration.

It is also worth noting that no previous review in this period explicitly addresses all three modalities—imaging, respiratory acoustics, and EHR data—within a single comparative framework. Reviews focusing on multimodal learning in general medicine [17] or on COVID-19 diagnosis [20,21] touch on subsets of this space, but their scope either extends beyond pulmonary medicine or restricts itself to a single-disease episode. Our review occupies a gap: the intersection of multimodal methodology and the full spectrum of major pulmonary pathologies within a bounded and current timeframe.

### 5.5. Limitations of This Review

Several caveats deserve explicit mention. First, this is a narrative review, not a systematic one: no protocol was pre-registered, and results were not pooled meta-analytically; however, a structured methodological quality assessment of the key included studies, covering dataset provenance, external validation, and publication type, is now provided in Table 6 (Section 5.3). Because the studies included span heterogeneous datasets, evaluation metrics and disease categories, a formal meta-analysis was neither feasible nor the stated objective; the review should be read as a synthesis of the state of the art rather than as an evidence base for clinical decision-making. Second, the approximate study counts reported in Section 2 reflect narrative selection rather than a fully documented PRISMA cascade; the absence of a complete per-stage exclusion count is a limitation we acknowledge. Third, we restricted ourselves to English-language publications, which inevitably omits a meaningful fraction of respiratory AI work, particularly from China and Latin America, where output in this area has grown substantially. Fourth, performance numbers are those reported by the original authors on their own test sets; we did not independently replicate or validate any model, so selection bias, dataset overlap between training and test splits, and evaluation–protocol differences all carry through to our synthesis. Fifth, a small number of preprints were retained where no peer-reviewed equivalent was available; those entries are flagged in the reference list and should be treated as tentative. Finally, the search was closed on 31 December 2024, and the field has continued to advance since then.

### 5.6. Implications for Future Research

Looking forward, three lines of work strike us as the most consequential. The first is benchmarks. The field badly needs public datasets that pair imaging, respiratory sounds, and EHR data for the same patient cohort, balanced across age, sex, and ethnicity, and labeled with conventions that the community has actually agreed on, particularly for adventitious sounds, where current annotation practices vary even between adjacent papers. The second is foundation models for medical audio. Wav2Vec 2.0 and HuBERT transformed how speech is handled; there is no obvious reason the same recipe, applied to large unlabeled corpora of lung sounds, would not yield comparable gains, and it would directly relieve the annotation bottleneck that currently constrains the field. The third is methodological: fairness and interpretability cannot remain afterthoughts. Given the now well-documented underdiagnosis of marginalized groups by current chest-X-ray models, reporting subgroup performance and explanation quality alongside aggregate accuracy should be table stakes, not a bonus.

## 6. Conclusions

Clinical reasoning has always been multimodal. A physician integrates what she sees on the radiograph, hears through the stethoscope, reads in the chart, and gathers from the patient in front of her, and the diagnosis emerges from the combination, and not from any one channel. The deep learning literature reviewed here is, in a sense, finally catching up with that fact. Models that fuse imaging, respiratory acoustics, clinical text, laboratory values, and epidemiological context are starting to outperform their single-modality predecessors by margins that matter clinically, not just statistically. Two architectural ideas drive most of the progress we documented. The first is hybridization: convolutional, attention-based, and self-supervised components stacked or combined within a single pipeline. The second is self-supervised pre-training, which has done for medical audio what it earlier did for natural language, turning the scarcity of labeled data from a hard ceiling into a soft one. Acoustic models, in particular, are no longer a curiosity. They classify coughs and respiratory sounds with clinically useful accuracy; they need only a microphone, and they fit the telemedicine and resource-limited contexts where a CT scanner is not an option. What is missing is less a matter of cleverer models than of infrastructure. The field needs multimodal datasets that pair imaging, sound, and EHR data for the same patients and that are balanced across the populations the models will eventually serve. It needs foundation models trained specifically on medical audio and imaging at scale. And it needs to stop treating fairness, interpretability, and interoperability—HL7/FHIR compliance among them—as nice-to-haves that come after the accuracy number is published. None of this is technically out of reach. It is, in our view, mostly a question of where the community decides to put its effort over the next few years. That decision is what will determine whether multimodal deep learning for lung disease becomes another impressive benchmark, or an actual clinical tool.

## Figures and Tables

**Table 1:** Performance comparison of key CNN models for lung disease detection (X-ray/CT); sources are cited per row.

Study/Model Name	Dataset Used	Method/Architecture	Key Performance Metrica *
Dardouri et al.	Kaggle chest X-ray dataset	Customized CNN+	97.24%
	(6400 images)	Adam optimizer	
LCP-CNN	UK hospitals (1397 pulmonary nodules)	Convolutional neural network (CNN)	AUC 89.6%
Xception	Kaggle’s pneumonia detection dataset	CNN	96%
CNN	5400 CXR images	CNN	96%
VisionTransformer (ViT)	Chest X-ray image dataset (19,003 images)	ViT-based deep learning	95.87%
EfficientNetB0	Guangzhou Women and Children’s Medical Center	EfficientNetB0	95.19%
VGG19	Kaggle’s pneumonia detection dataset	CNN	95%
VGG16	Kaggle’s pneumonia detection dataset	CNN	95.4%
YOLOv3	Curated COVID-19 CXR images	YOLOv3	92.15%
CNN	More than 5000 real images	CNN	84%

* Values correspond to classification accuracy, except for LCP-CNN, for which the original study reports AUC.

**Table 2:** Comparative accuracy of acoustic feature types for abnormal symptom detection.

Feature Type	Classification Accuracy (%)
Mel spectrogram	97.13%
Spectrogram	95.22%
MFCC	93.78%

**Table 3:** Performance benchmarks of temporal deep learning models for lung sound analysis [39,40].

Model Variant	Key Performance	Characteristics
GRU-based models	Generally higher F1 scores (0.7–9.5% higher) and AUC values in most tasks	Computationally more efficient
Bidirectional models (BiLSTM, BiGRU)	Consistently outperformed unidirectional counterparts (F1 0.4–9.8% higher)	Effective at capturing comprehensive context
Models with CNN	Outperformed in 26 out of 32 pairs; significant improvement for CAS detection (F1 26–30.3% higher, AUC 0.067–0.089 higher)	Effective at extracting local positional information
Audio Spectrogram Transformers (ASTs) with audio enhancement	On ICBHI, sensitivity increased by 11.71 percentage points for fine-tuned AST and by 17.08 percentage points for Patch-Mix AST; ICBHI score increased by 1.40 and 1.60 points, respectively	Leverage pre-trained models and Patch-Mix for performance and overfitting prevention

**Table 4:** Comparative summary of public chest X-ray (CXR) and CT datasets used in deep learning for lung disease detection.

Dataset	Primary Modality	Number of Labels	Total Images
ChestX-ray14	CXR	14 disease labels	112,120
CheXpert [53]	CXR	14 observations	224,316
COVIDx	CXR	Variety of labels	33,920
LUNA16	CT	36,378 annotations	888

**Table 5:** Comparative analysis of public acoustic datasets.

Dataset	Total Duration	Sound Types (Primary)	Diseases	Challenges
ICBHI	5.5 h	Breathing	Pneumonia, LRTI, Asthma, Bronchiectasis, URTI, Bronchiolitis, COPD	Signal variability (different equipment and sampling rates); noise
Coswara	65 h	Breathing, cough	SARS-CoV-2 (negative, positive, recovered), respiratory ailments	Primarily from India (geographic bias); demographic and language bias analysis needed
AudioCOVID	N.E.	Human speech	COVID-19, pneumonia, COPD, other respiratory diseases	Noise; variability
HF_Lung_V1	Expanded to HF_Lung_V2 (1.43 × increase in audio files)	Breath phase (inhalation, exhalation), adventitious sounds (CAS, DAS)	N.E.	Noisy labels; overlapping sounds

## Data Availability

No new data were created or analyzed in this study.

## Supplementary Materials

The following supporting information can be downloaded at: https://www.mdpi.com/article/10.3390/medicina62071223/s1.
