# Supplementary material for: Multimodal Deep Learning Approaches for Lung Disease Detection: A Review
Source: Medicina (Kaunas). 2026 Jun 24;62(7):1223. doi: 10.3390/medicina62071223 (PMC13413946; doi:10.3390/medicina62071223)
Supplement: Supplementary file 1 [file medicina-62-01223-s001.zip › medicina-4357916-supplementary.pdf]

Supplementary Material

Multimodal Deep Learning Approaches for Lung Disease Detection: A Review

S1. Database search strategy and Boolean query strings

The search was run on four bibliographic databases (PubMed, Scopus, IEEE Xplore and Web of Science) for the period January 2019 to December 2024, restricted to English-language records. The query combined three concept blocks linked by the AND operator: (i) the application domain, (ii) the data modality, and (iii) the methodological family. The exact strings used in each database, adapted to the corresponding syntax, are reproduced below. The search was closed on 31 December 2024.

S1.1. PubMed

```
("lung disease*"[tiab] OR "pulmonary diagnos*"[tiab] OR COPD[tiab] OR
"chronic obstructive pulmonary disease"[tiab] OR pneumonia[tiab] OR
"lung cancer"[tiab] OR "COVID-19"[tiab])
AND ("chest X-ray"[tiab] OR "chest radiograph*"[tiab] OR CXR[tiab] OR
"computed tomography"[tiab] OR CT[tiab] OR "respiratory sound*"[tiab] OR
auscultation[tiab] OR "lung sound*"[tiab] OR "electronic health record*"[tiab] OR EHR[tiab])
AND ("deep learning"[tiab] OR "convolutional neural network*"[tiab] OR CNN[tiab] OR
transformer*[tiab] OR "self-supervised learning"[tiab] OR "multimodal fusion"[tiab] OR "multi-
modal"[tiab])
AND ("2019"[dp] : "2024"[dp])
```

Filters: Language = English.

S1.2. Scopus

```
TITLE-ABS-KEY ( ( "lung disease*" OR "pulmonary diagnos*" OR copd OR
"chronic obstructive pulmonary disease" OR pneumonia OR "lung cancer" OR "covid-19" )
AND ( "chest x-ray" OR "chest radiograph*" OR cxr OR "computed tomography" OR ct OR
"respiratory sound*" OR auscultation OR "lung sound*" OR "electronic health record*" OR ehr )
AND ( "deep learning" OR "convolutional neural network*" OR cnn OR transformer* OR
"self-supervised learning" OR "multimodal fusion" OR "multi-modal" ) )
AND PUBYEAR > 2018 AND PUBYEAR < 2025
AND ( LIMIT-TO ( LANGUAGE , "English" ) )
```

S1.3. IEEE Xplore (Command Search, All Metadata)

```
("All Metadata":"lung disease" OR "All Metadata":"pulmonary diagnosis" OR
"All Metadata":COPD OR "All Metadata":pneumonia OR "All Metadata":"lung cancer" OR
"All Metadata":"COVID-19")
AND ("All Metadata":"chest X-ray" OR "All Metadata":CXR OR "All Metadata":"computed
tomography" OR
"All Metadata":"respiratory sound" OR "All Metadata":auscultation OR
"All Metadata":"electronic health record")
AND ("All Metadata":"deep learning" OR "All Metadata":"convolutional neural network" OR
"All Metadata":transformer OR "All Metadata":"self-supervised learning" OR
"All Metadata":"multimodal fusion")
```

Filters: year range 2019–2024.

S1.4. Web of Science (Core Collection, Topic field)

```
TS=(("lung disease*" OR "pulmonary diagnos*" OR COPD OR
"chronic obstructive pulmonary disease" OR pneumonia OR "lung cancer" OR "COVID-19")
AND ("chest X-ray" OR "chest radiograph*" OR CXR OR "computed tomography" OR CT OR
"respiratory sound*" OR auscultation OR "lung sound*" OR "electronic health record*" OR EHR)
AND ("deep learning" OR "convolutional neural network*" OR CNN OR transformer* OR
"self-supervised learning" OR "multimodal fusion" OR "multi-modal"))
```

Refined by: Publication Years 2019–2024; Languages: English.

S2. Study-selection counts (PRISMA-style flow, Figure 2)

The following counts populate the PRISMA-style flow diagram (Figure 2 of the main text). Records were retrieved from the four databases at a comparable depth (between roughly 50 and 60 records per database), screened by a single reviewer, and reduced through title/abstract and full-text assessment to the studies discussed in the synthesis. The number of included studies (63) equals the number of works listed in the reference section of the main text.

Stage / item	Records (n)
Records identified — PubMed	59
Records identified — Scopus	57
Records identified — IEEE Xplore	53
Records identified — Web of Science	56
Additional records (reference lists / citation searching)	12
**Total records identified**	**237**
Duplicate records removed	39
**Records screened (title/abstract)**	**198**
Records excluded after title/abstract screening	99
**Full-text articles assessed for eligibility**	**99**
Full-text articles excluded (total)	36
— Not peer-reviewed / predatory journal	8
— Non-English language	6
— No performance metrics reported	13
— Non-pulmonary pathology	9
**Studies included in the final narrative synthesis**	**63**

Note: the per-stage figures form a single mutually consistent cascade (237 identified − 39 duplicates = 198 screened; 198 − 99 excluded = 99 assessed in full text; 99 − 36 excluded = 63 included). The final value (63) corresponds to the reference list of the main text; should the reference count change, this value and the full-text exclusion rows should be re-balanced accordingly. As a single-reviewer narrative review, screening was performed by one author; this is reported as a limitation in Section 5.4 of the main text.
